# Supplementary material for: Association of the interatrial block and left atrial fibrosis in the patients without history of atrial fibrillation
Source: PLoS One. 2024 Feb 8;19(2):e0297920. doi: 10.1371/journal.pone.0297920 (PMC10852280; doi:10.1371/journal.pone.0297920)
Supplement: S1 File — (PDF) [file pone.0297920.s001.pdf]

Research Proposal

1. Title of the project

Association of LA abnormality by ECG and left atrial scar from cardiac MRI

2. Research objectives

Aim of this study is to assess the correlation of LA disorders by ECG such as interatrial block, LA abnormality and LA scar in patients undergoing cardiac magnetic resonance imaging.

3. Background or Literature review

Left atrial abnormality is associated with significant cardiovascular morbidity and mortality.(1) Strong evidence suggests that, increased left atrial size has been associated with severity and duration of atrial fibrillation. LA abnormality is also a predictor of stroke once atrial fibrillation is manifest. It has been associated with decreased survival after myocardial infarction, congestive heart failure, and reduction in left ventricular ejection fraction.(2, 3) Detection of LA abnormality since asymptomatic may lead to early detection of those cardiac conditions with prompt appropriate therapy accordingly.

Electrical and structural remodeling of the atrium occurs in patients with atrial fibrillation (AF) and structural heart disease. (4, 5) In particular, studies have demonstrated that some patients with atrial arrhythmias have spontaneous atrial scarring characterized by discrete regions of low voltage.(4, 6) Left atrial scarring (LAS) may serve as a substrate for slow conduction and intra-atrial re-entry, which may predispose to future atrial arrhythmia.(4, 7, 8)

Interatrial block (IAB) is defined as prolonged conduction time between the RA and LA due to impulse delay or blockage, probably most often but not exclusively in the Bachmann bundle (BB), resulting in prolonged P-wave duration (≥120 milliseconds) .(9, 10)

In analogy to other conduction delays, IAB may be graded as first, second and third degrees or partial and advanced IAB.(10-12) Partial IAB is synonymous to first degree IAB whereas the term advanced IAB encompasses both second and third degree IAB. The distinction is based on the P-wave duration and more importantly, the P-wave morphology. IAB with bifid P wave (notched P-

wave) in leads I, II, III and aVF, is considered to represent partial or first degree IAB, as they represent normal propagation with a conduction delay. P wave morphology in V1 in partial IAB often presents with a negative mode or a biphasic mode where negative phase is less evident than in cases of associated left atrial enlargement (LAE). (10, 11) Third degree IAB refers to those with biphasic P waves (± P-waves) in inferior leads (II, III and aVF), indicating caudo-cranial left atrial activation, usually due to a fixed block in the normal route of conduction. (11, 13) The positive mode of P-waves in leads II, III and aVF may sometimes not be well seen because of underlying fibrosis and the diagnosis of junctional rhythm due to an apparently negative P wave in inferior leads may be made. (10, 11) IAB are frequently associated to LAE that, in the case of third-degree interatrial blocks, have a specificity of 90% and to supraventricular arrhythmias that are also, much more frequent in third-degree interatrial blocks. The prevalence of first-degree interatrial block in the general population is very high, whereas third-degree interatrial blocks are rarely seen. (10) The prevalence of IAB has been shown to increase with age with only 9% in those less than 35 years,(14) to 40-60% at ages over 50 years. (11, 15-17)

Delayed enhancement MRI (DE-MRI) using gadolinium contrast has been demonstrated to localize and quantify the degree of structural remodeling or fibrosis associated with AF in the LA. (18, 19) DE-MRI has also been shown to be useful in localizing and quantifying scar formation in the LA following radiofrequency ablation (RFA). (18, 20, 21)

There have not been previous studies that examine the correlation of ECG criteria of LA abnormality and left atrial scar from Cardiac MRI. Therefore, this study aimed to assess the association of ECG criteria to diagnose LA abnormality and left atrial scar.

4. Rationale

Cardiac MRI (CMR) is another imaging technique that has been increasingly used to assess cardiac chamber size. CMR can provide more precise volumetric assessment of cardiac chamber size and have less inter-observer variability of measurements compared to echocardiography. However, CMR is also not widely available, is expensive, require higher technical expertise, take longer time. Correlation of ECG criteria of LA assessment (partial IAB, advance IAB, LA abnormality) and left atrial scar from Cardiac MRI may be useful applied to clinical practice.

5. Research methodology

5.1 Outline of study plan including methodology

This is a retrospective analysis of patients presenting to the Maharaj Nakhon Chiang Mai hospital for Cardiac MRI during the study periods.

The inclusion criteria were

(1) Age>18 years

(2) Patients undergoing cardiac magnetic resonance imaging (CMR) between January 2013 to December 2020

(3) ECG 12 lead was performed.

Patients with history of chronic AF, sinus arrest, Atrio-ventricular block were excluded. Demographic characteristics were collected. The study protocol was approved by the ethics committee of institution.

<u>Definitions</u>

ECG criteria for LA abnormality: At least 1 criteria from the followings;

- P wave in lead II, III, aVF > 120 ms
- Biphasic P wave in V1 by P terminal force > 40 ms

ECG criteria for Partial IAB was defined as a P-wave duration of >120 ms

ECG criteria for advanced IAB was defined as a P wave of >120 ms and biphasic morphology (±) in the inferior leads.

ECG 12-lead (filter 150 Hz, 25 mm/s, 10 mm/mV) was analyzed by the ECG range measurement program.

Cardiac magnetic resonance imaging (CMR)

Data of CMR between 1 April 2013 to 31 May 2020 was collected from medical information. CMR images were manually segmented to isolate the LA to quantify the spatial extent of delayed enhancement ie.atrial scar.

Atrial fibrosis or scar divided into 2 Major categories.

The first major categories divided into 5 grading; grade 0: no atrial scar, grade 1: atrial scar<25%, grade 2: atrial scar 25 to 50%, grade 3: atrial scar 51 to 75%, and grade 4: atrial scar more than 75%.

The second major categories divided into 2 grades: atrial scar less than 50% and atrial scar more than 50%.

ECG and CMR analyses were interpreted by 4 experienced investigators blinded to other results.

5.2 Method of data analysis

Continuous variables are expressed as mean ± SD after verification of the normal distribution. Categorical data were summarized as frequencies and percentages. Univariate comparisons (independent samples t-tests and $\chi 2$-tests). A p value < 0.05 was considered significant. Statistical analysis was performed using statistical software (SPSS version 10.0).

Sample size calculation

Estimate 230 patients included in this study according to number of patients who underwent cardiac MRI during timing of retrospective analysis in our hospital (January 2013 to December 2020).

5.3 Time Table

Activities	July – August 2021	September 2021	October 2021 – March 2022	April – May 2022	June 2022
1. Draft research project and review literature	√				
2. Propose ethical committee		√			
3. Data collection			√		
4. Data analysis				√	
5. Reporting					√

6. Reference

1. Bombelli M, Cuspidi C, Facchetti R, Sala C, Tadic M, Brambilla G, et al. New-onset left atrial enlargement in a general population. Journal of hypertension. 2016;34(9):1838-45.

2. Bureekam S, Boonyasirinant T. Accuracy of left atrial enlargement diagnosed by electrocardiography as compared to cardiac magnetic resonance in hypertensive patients. Journal of the Medical Association of Thailand = Chotmaihet thangphaet. 2014;97 Suppl 3:S132-8.

3. Tsao CW, Josephson ME, Hauser TH, O'Halloran TD, Agarwal A, Manning WJ, et al. Accuracy of electrocardiographic criteria for atrial enlargement: validation with cardiovascular magnetic resonance. Journal of cardiovascular magnetic resonance : official journal of the Society for Cardiovascular Magnetic Resonance. 2008;10:7.

4. Verma A, Wazni OM, Marrouche NF, Martin DO, Kilicaslan F, Minor S, et al. Pre-existent left atrial scarring in patients undergoing pulmonary vein antrum isolation: an independent predictor of procedural failure. Journal of the American College of Cardiology. 2005;45(2):285-92.

5. Wijffels MC, Kirchhof CJ, Dorland R, Allessie MA. Atrial fibrillation begets atrial fibrillation. A study in awake chronically instrumented goats. Circulation. 1995;92(7):1954-68.

6. Sanders P, Morton JB, Davidson NC, Spence SJ, Vohra JK, Sparks PB, et al. Electrical remodeling of the atria in congestive heart failure: electrophysiological and electroanatomic mapping in humans. Circulation. 2003;108(12):1461-8.

7. Li D, Fareh S, Leung TK, Nattel S. Promotion of atrial fibrillation by heart failure in dogs: atrial remodeling of a different sort. Circulation. 1999;100(1):87-95.

8. Goette A, Juenemann G, Peters B, Klein HU, Roessner A, Huth C, et al. Determinants and consequences of atrial fibrosis in patients undergoing open heart surgery. Cardiovascular research. 2002;54(2):390-6.

9. Mehrzad R, Spodick DH. Interatrial block: a virtual pandemic requiring attention. Iranian journal of medical sciences. 2014;39(2):84-93.

10. Bayes de Luna A, Platonov P, Cosio FG, Cygankiewicz I, Pastore C, Baranowski R, et al. Interatrial blocks. A separate entity from left atrial enlargement: a consensus report. Journal of electrocardiology. 2012;45(5):445-51.

11. Chhabra L, Devadoss R, Chaubey VK, Spodick DH. Interatrial block in the modern era. Current cardiology reviews. 2014;10(3):181-9.

12. Kitkungvan D, Spodick DH. Interatrial block: is it time for more attention? Journal of electrocardiology. 2009;42(6):687-92.

13. Bayes de Luna A, Guindo J, Vinolas X, Martinez-Rubio A, Oter R, Bayes-Genis A. Third-degree inter-atrial block and supraventricular tachyarrhythmias. Europace : European pacing, arrhythmias, and cardiac electrophysiology : journal of the working groups on cardiac pacing, arrhythmias, and cardiac cellular electrophysiology of the European Society of Cardiology. 1999;1(1):43-6.

14. Gialafos E, Psaltopoulou T, Papaioannou TG, Synetos A, Dilaveris P, Andrikopoulos G, et al. Prevalence of interatrial block in young healthy men<35 years of age. The American journal of cardiology. 2007;100(6):995-7.

15. Asad N, Spodick DH. Prevalence of interatrial block in a general hospital population. The American journal of cardiology. 2003;91(5):609-10.

16. Jairath UC, Spodick DH. Exceptional prevalence of interatrial block in a general hospital population. Clinical cardiology. 2001;24(8):548-50.

17. Ariyarajah V, Asad N, Tandar A, Spodick DH. Interatrial block: pandemic prevalence, significance, and diagnosis. Chest. 2005;128(2):970-5.

18. Akoum N, Daccarett M, McGann C, Segerson N, Vergara G, Kuppahally S, et al. Atrial fibrosis helps select the appropriate patient and strategy in catheter ablation of atrial fibrillation: a DE-MRI guided approach. Journal of cardiovascular electrophysiology. 2011;22(1):16-22.

19. Oakes RS, Badger TJ, Kholmovski EG, Akoum N, Burgon NS, Fish EN, et al. Detection and quantification of left atrial structural remodeling with delayed-enhancement magnetic resonance imaging in patients with atrial fibrillation. Circulation. 2009;119(13):1758-67.

20. McGann CJ, Kholmovski EG, Oakes RS, Blauer JJ, Daccarett M, Segerson N, et al. New magnetic resonance imaging-based method for defining the extent of left atrial wall injury after the ablation of atrial fibrillation. Journal of the American College of Cardiology. 2008;52(15):1263-71.

21. Peters DC, Wylie JV, Hauser TH, Kissinger KV, Botnar RM, Essebag V, et al. Detection of pulmonary vein and left atrial scar after catheter ablation with three-dimensional navigator-gated delayed enhancement MR imaging: initial experience. Radiology. 2007;243(3):690-5.

7. Expected Benefits from Research Project

Correlation of ECG criteria of LA assessment (partial IAB, advance IAB, LA abnormality) and left atrial scar from Cardiac MRI may be useful applied to clinical practice.

9. Investigator

9.1 Natnicha Pongbangli, MD

9.2 Wanwarang Wongcharoen, MD

9.3 Arintaya Phrommintikul, MD

9.4 Narawudt Prasertwitayakij, MD

9.5 Teerapat Nantsupawat, MD

9.6 Siriluck Gunaparn, RN
